# The Effects of Table Tennis on Motor Function and Cognitive Performance in Parkinson’s Disease

**DOI:** 10.7759/cureus.96383

**Published:** 2025-11-08

**Authors:** Alisa Khalid, Rayyan Khalid

**Affiliations:** 1 Medicine, University of Glasgow, Glasgow, GBR; 2 Trauma and Orthopaedics, University Hospital Crosshouse, Glasgow, GBR; 3 Medicine, University of Edinburgh, Edinburgh, GBR

**Keywords:** cognitive performance, exercise-based therapy, motor function recovery, neurorehabilitation neurorehab, parkinsons disease, table tennis therapy

## Abstract

Parkinson’s disease (PD) is a chronic, progressive neurodegenerative disorder with motor and cognitive impairments that significantly impact quality of life. Despite many advances in PD management, there is currently no disease-modifying or curative therapy available. Exercise-based rehabilitation promotes neuroplasticity and functional recovery through synaptogenesis, angiogenesis, and the release of neurotrophic factors. Exercise is increasingly utilised as a complementary strategy to pharmacological management in PD patients. This review investigates the therapeutic potential of table tennis as a physical exercise intervention for patients with PD. The motor, cognitive, and psychosocial effects are analysed from current literature. A comprehensive literature search was conducted across multiple electronic databases: Google Scholar, iDiscover, PubMed, and Cochrane Library up to October 2025. Studies that were included investigated table tennis or a similar skill-based exercise therapy used in individuals with PD. The current evidence demonstrates improved motor performance, balance, coordination, and activities of daily living with table tennis therapy. Additionally, cognitive improvements were observed, including enhanced attention, visuospatial processing, and executive function. Psychosocial benefits, such as motivation, social engagement, and emotional well-being, were reported. Compared with conventional exercise therapy, table tennis utilised a unique combination of cognitive challenge, physical precision, and social interaction. This combination may enhance adherence and engagement to therapy. Most studies were limited by small sample sizes, short intervention duration, and non-standardised protocols. This restricted generalisability to the broader population. A multicentre randomised controlled trial with a long-term longitudinal study and objective outcome measure is required. To conclude, table tennis is a feasible, enjoyable, and multidimensional option for rehabilitation in PD. It can potentially complement existing rehabilitation for PD.

## Introduction and background

Parkinson’s disease (PD) is a chronic, progressive neurodegenerative disorder that primarily affects the dopaminergic neurons within the substantia nigra pars compacta of the basal ganglia. Despite advances in PD management, there is no current disease-modifying or curative therapy available [1,2]. In addition to pharmacological and device-aided therapies, rehabilitation is highly recommended to help patients maintain their activities of daily living [1,3]. Therefore, rehabilitation programmes have become essential in maintaining maximum mobility and independence in individuals with PD. Exercise, defined as a planned and structured physical activity, aims to improve physical fitness. There is growing evidence that the benefits of exercise include enhancing neuroplasticity and the ability of the brain to self-repair [4]. Rehabilitation exercises can be adapted to various locations, are straightforward to conduct, and can be tailored to the severity of each individual’s condition. There is evidence that supports the effects of exercise for patients with PD with regard to physical functioning, quality of life, balance, coordination, and strength [5]. Numerous physical interventions have already been introduced in rehabilitation with PD patients. Some of these physical interventions include tai chi [6], Lee Silverman Voice Treatment (LSVT) BIG [7], robot-assisted gait training [8], boxing [9], and dance therapy [10]. Zigmond and Smeyne [11] concluded that exercise offered neuroprotection by increasing mitochondrial energy production, reducing inflammation, stimulating antioxidant activity, angiogenesis, and producing synaptogenesis.

Table tennis is a racket sport regularly played across the world with approximately 300 million recreational players and 40 million competitive players [12]. The rules of the game are uncomplicated, and physical requirements are minimal. The sport requires a high level of hand-eye coordination and concentration in order to predict and react to various trajectories of the ball [13]. Table tennis involves few injuries as it can be played in accordance with age, physical strength, and skill. Evidence suggests that table tennis may enhance cognitive function more effectively than other forms of exercise. Table tennis has been reported to improve hand-eye coordination, mental acuity, balance, reflexes, and physical strength [14]. It has also proven beneficial as a social outlet and for emotional well-being. In PD, the depletion of dopamine disrupts the balance between the excitatory and inhibitory pathways, which regulate voluntary movement. This neurochemical imbalance manifests clinically as resting tremor, rigidity, postural instability, and bradykinesia [15]. Table tennis has been explored as a therapeutic intervention for individuals with PD to improve motor performance, balance, and cognitive function [16]. The sport demands fast-paced visual-motor responses, fine motor precision, and anticipatory control; this stimulates both motor and cognitive networks, which can be compromised in PD. Additionally, the social aspect of the sport may enhance adherence and overall well-being [15]. However, current evidence surrounding this is limited by methodological variability and small sample size.

This review aims to synthesise and critically appraise the current literature that analyses the therapeutic potential of table tennis as an exercise-based intervention for individuals with PD.

## Review

Methods

A comprehensive literature review was conducted by two researchers to identify and evaluate studies exploring the effects of table tennis on motor and cognitive performance in individuals with PD. Uncertainties regarding study eligibility were resolved through discussion and consensus. The review followed the general principles of the Preferred Reporting Items for Systematic Reviews and Meta-Analysis (PRISMA) framework to ensure methodological transparency and reproducibility. Formal protocol registration was not performed. 

Two independent researchers (author and co-author) screened titles and abstracts for relevance. This was followed by a full-text assessment of potentially eligible studies. Disagreements were resolved by discussion and consensus. The data extracted from the literature involved: study, author’s name, publication year, study design, and findings. 

A comprehensive search was conducted across multiple electronic databases. The following databases were used for the literature review: Google Scholar, iDiscover, PubMed, and Cochrane Library. The search was conducted up to October 2025. A combination of the following keywords was used: “Parkinson’s disease”, “Parkinsonism”, “table tennis”, “exercise”, “ping pong”, “physical activity”, “physical therapy”, “cognition”, “motor function”, “older adults”, ”elderly”. Additional sources were identified by manually screening the reference lists of relevant articles and reviews. 

Literature selection was based on the evidence-based medicine PICOS (participants, intervention, comparison, outcome, study design) framework. The following inclusion criteria were considered to select literature: participants diagnosed with PD, intervention involved table tennis or exercise-based programmes, and outcomes related to motor function, coordination, balance, or cognitive performance were reported. 

Exclusion criteria were applied to articles that did not meet the inclusion criteria, were not available in English, or focused on non-Parkinsonian populations. The studies that were selected based on the inclusion criteria are displayed in Table 1.

**Table 1:** Studies Selected Based on Inclusion Criteria

Study	Author	Year	Study Design	Findings
Tai chi and postural stability in patients with Parkinson's disease	Li et al. [6]	2012	Randomised Controlled Trial	Tai chi training reduces balance impairments in individuals with mild-to-moderate Parkinson's disease. There was also improved functional capacity and reduced falls noted.
Effect of exercise on motor and nonmotor symptoms of Parkinson's disease	Dashtipour et al. [7]	2015	Randomised Controlled Trial	Results concluded there were positive effects of general exercise and Lee Silverman Voice Therapy BIG on motor and non-motor symptoms of Parkinson's disease. General exercise could be just as effective and useful for patients unable to access outpatient Lee Silverman Voice Therapy BIG.
Effects of robot-assisted gait training in patients with Parkinson's disease: study protocol for a randomized controlled trial	Kang et al. [8]	2019	Randomised Controlled Trial	Robot-assisted gait training with external cues or feedback is expected to improve gait performance in patients with Parkinson's disease.
Boxing training for patients with Parkinson's Disease: a case series	Combs et al. [9]	2011	Case Series	Patients displayed short-term and long-term improvements in balance, gait, activities of daily living and quality of life following the boxing training program.
Dance therapy improves motor and cognitive functions in patients with Parkinson's disease	de Natale et al. [10]	2017	Quasi- Experimental Study	Dance therapy was found to improve motor (endurance and risk of falls) and non-motor functions (executive functions). These improvements were still evident after long-term follow-up.
Table tennis for health: a multidimensional perspective on its physical, emotional, and social advantages	Aparicio-Chueca and Muñoz-Vila [14]	2025	Mixed Method	Table tennis is a low-impact exercise that can enhance physical, emotional, and social well-being. It is accessible, adaptable, and it is appropriate for diverse populations.
A pilot study of the feasibility and effects of table tennis training in Parkinson's disease	Olsson et al. [15]	2020	Single Group Observational Study	Table tennis training is a safe and feasible sport. It may have the potential to improve balance control, mental well-being, and physical activity levels. Further research is required.
A retrospective comparison of physical health in regular recreational table tennis participants and sedentary elderly men	Naderi et al. [17]	2018	Randomised Controlled Trial	The outcomes suggest that regular table tennis has advantageous effects on muscle strength, physical performance, and body composition. It is a form of exercise that can improve health in older adults.
Table tennis for patients with Parkinson's disease: a single-center, prospective pilot study	Inoue et al. [18]	2021	Prospective Cohort Study	Table tennis is a safe form of exercise rehabilitation and may improve activities of daily living and motor symptoms in individuals with PD.

Due to the limited number of studies and heterogeneity in methodology, sample size, and outcome measures, a qualitative synthesis was performed as opposed to a meta-analysis. The findings were grouped under two primary domains - motor performance outcomes and cognitive outcomes. Where applicable, secondary observations were noted, such as motivation, mood, adherence, social, and emotional outcomes. 

Ethical approval was not required as this study involved a review of existing literature. All studies that were reviewed and considered were assumed to have adhered to local ethical standards and obtained appropriate participant consent.

Discussion

Growing evidence supports exercise as a therapeutic intervention for motor and non-motor symptoms in PD. Table tennis has emerged as a rehabilitation method that offers a unique combination of cognitive, motor, and psychosocial benefits. The review synthesises current evidence on the effects of table tennis in PD as a form of exercise-based rehabilitation and outlines limitations and future directions for research.

Current Exercise Interventions in Parkinson’s Disease

Exercise is an established adjunct to pharmacological and device-aided therapies in PD management. Systematic reviews provide evidence that physical activity may improve motor performance and quality of life in individuals with PD [16]. However, there are uncertainties surrounding optimal intensity, frequency, and duration of exercise interventions. Randomised control trials have demonstrated how general exercise programmes may be as effective as LSVT BIG therapy in improving motor and non-motor outcomes [7]. Another study compared LSVT BIG therapy with Nordic walking and at-home general exercises; these interventions focused on repetitive, high-intensity, and complex movements [19]. The study found that quality of life or motor skills did not significantly differ between the two groups following therapy. This suggests that accessibility, patient preference, and long-term sustainability of exercise could be equally important determinants of therapeutic success. 

Li et al. [6] investigated the effects of Tai Chi in patients with mild-moderate PD and reported improvements in postural stability, functional capacity, and reduced falls. Dance therapy has likewise improved motor and cognitive functions [10]. de Natale et al. [10] reported that dance sessions improved gait, balance, and executive function. It is also known to engage participants socially and promote emotional expression. Dance has a rhythmic and synchronised approach which can stimulate the basal ganglia and facilitate sensorimotor integration. Boxing is another form of exercise used for rehabilitation, such as non-contact boxing training in PD [9]. Boxing has been shown to improve mobility, balance, and endurance. In a case series, Combs et al. [9] demonstrated that participants undertaking a structured boxing regimen displayed improved postural stability, gait velocity, and confidence in activities of daily living. Boxing creates an environment that is high intensity and goal-directed; this can reinforce neural plasticity and motor learning through continuous engagement of proprioceptive and visuomotor pathways.

Inoue et al. [18,20] explained that exercise has the potential to show neuroprotective properties through processes such as an increase in mitochondrial energy production, antioxidant activity, reduced inflammation, angiogenesis, and synaptogenesis [20,21]. This theory provides a foundation for why skill-based sports such as table tennis might engage numerous motor and cognitive circuits simultaneously. Moreover, Ellis et al. [22] found that early exercise interventions were crucial to slowing functional decline, preventing irreversible motor decline, and preserving mobility. These findings reinforce the importance of introducing an accessible and sustainable exercise intervention, such as table tennis, at an early stage of PD to maximise the potential of neuroprotection and functional independence.

Table Tennis as a Therapeutic Exercise

Recent evidence indicates growing interest in table tennis as a rehabilitation tool for PD. A series of prospective and pilot studies have explored its feasibility and potential effects on motor, cognitive, and psychosocial outcomes [15,18]. Inoue et al. [18] conducted a six-month prospective study and reported that regular participation in a structured table tennis training programme was associated with improved motor symptoms and activities of daily living. The study also found that table tennis participation led to enhancements in balance control, emotional well-being, and physical activity levels. Both Olsson et al. and Inoue et al. highlighted the low-impact, accessible, and engaging nature of table tennis, which may enhance adherence to exercise rehabilitation in older adults with PD [15,18]. 

Importantly, programmes incorporated safety adaptations to minimise the fall risk associated with PD patients in table tennis sessions. Adaptations included supervised sessions, slower-paced rallies, and the option for supported or seated play. This demonstrates that, with appropriate supervision and modification, table tennis can be a safe and feasible component of PD rehabilitation.

Moreover, a single-centre prospective trial similarly reported that participation in table tennis sessions produced significant improvement in motor performance and cognitive processing. These outcomes were derived from relatively small sample sizes; however, the evidence collectively suggests the feasibility and therapeutic benefit of incorporating table tennis into PD rehabilitation programmes. 

Local initiatives such as “Ping Pong Parkinson” have already introduced a competitive element to table tennis rehabilitation in PD [23]. Parkinson’s UK [24] has recognised the benefits of table tennis as rehabilitation and guides how to get involved in Parkinson’s only table tennis sessions. 

Mechanism of Therapeutic Action

The therapeutic benefits of table tennis likely arise from several neurobiological mechanisms. Studies highlighted that exercise enhances neuroplasticity by increasing mitochondrial activity, angiogenesis, and synaptogenesis [18,21]. Cortical and subcortical motor loops may be further engaged through the repetitive movements required in table tennis, facilitating compensatory neural activity in the cerebellum, motor cortex, and basal ganglia. The continuous demand for sensory prediction and motor correction can strengthen connections within the frontoparietal and visuomotor networks - systems often impaired in PD. 

Evidence from functional neuroimaging indicates cognitively engaging physical activities may enhance cortical efficiency and are hypothesised to influence dopamine release and related neural processes [23]. Table tennis can elicit these adaptive responses to aid enhancement in motor and cognitive reserves [25,26]. Table tennis can have the potential to be a neurorestorative intervention rather than a symptomatic therapy solely through its combination of aerobic exertion, fine motor precision, and cognitive engagement [15]. However, current studies investigating table tennis as a form of rehabilitation in PD do not incorporate the use of measured outcomes. Neuroimaging - already used to monitor PD progression [27] - could serve as a potential outcome-measuring tool for future table tennis intervention studies in PD.

Motor Outcomes

Table tennis demands rapid, repetitive, and precise upper and lower limb movements - these movements stimulate motor learning. PD involves the degeneration of dopaminergic neurons in the substantia nigra. This impairs basal ganglia function, which results in clinical manifestations such as bradykinesia, rigidity, and postural instability. The repetitive and rhythmic nature of table tennis could reinforce compensatory neural circuits through the mechanism of experience-dependent plasticity [27]. 

Yamasaki [12] investigated the biomechanics of table tennis and found that a high level of whole-body coordination and footwork was required to maintain stability and agility. These characteristics of the sport make it suited to addressing gait and balance concerns, which are common in PD. The motor outcomes reported by Inoue et al. [18] involved improvements in the Unified Parkinson’s Disease Rating Scale (UPDRS) scores, which comprised better dynamic balance and reduced tremor. The studies conducted were preliminary, although their results corroborate similar benefits that have been observed in other exercise-based regimes such as boxing and dance. Boxing and dance are also known to promote coordination, rhythm, and proprioceptive feedback.

Cognitive Outcomes 

Alongside the motor improvements, table tennis encourages, there may be significant cognitive benefits. The sport is well-known to require anticipatory control, sustained attention, visuospatial processing, and rapid decision-making; all cognitive domains that can be impaired in PD [14]. Recently conducted systematic reviews have encompassed studies on table tennis in neurodegenerative conditions such as PD, Alzheimer’s disease, and other types of dementia [23, 26]. These reviews concluded that table tennis is a safe, feasible, and effective non-pharmacological intervention to enhance cognitive and motor outcomes. The dual-task demands of table tennis, rapid sensory input processing, and immediate motor output, can strengthen cortical networks and executive function integration. 

Systematic reviews [28,29] looked at exercise and cognitive function in older adults. These reviews found that regular physical activity has evidence to show improvements in attention, memory, and executive functioning. Increased cerebral blood flow, neurogenesis, and modulation of neurotrophic factors may mediate these cognitive effects. Table tennis may enhance these mechanisms similarly. As the sport engages rapid perceptual-motor processing, it is a cognitively stimulating exercise - particularly useful for neurodegenerative populations. 

Psychosocial and Emotional Outcomes

Social and emotional benefits from table tennis are another important aspect of its therapeutic mechanism. Recent reviews conducted by Aparicio-Chueca et al. [14], Łosińska and Maszczyk [25], and Marta et al. [26] support table tennis as an inclusive, low-impact exercise capable of encouraging emotional well-being, sociability, and motivation among players across a variety of ages and abilities. The competitive and interactive nature of the sport can mitigate social isolation and depression. Social isolation and depression are prevalent in PD and have the potential to exacerbate disease burden. 

Moreover, a retrospective study by Naderi et al. [17] compared recreational table tennis players with sedentary older men. This study found that there was superior physical performance, muscle strength, and body composition among table tennis players. However, as the comparison group was sedentary, these differences may reflect the general benefits of regular physical activity rather than specifically table tennis. Nevertheless, the findings suggest recreational engagement in table tennis is still sufficient to contribute to physical and psychosocial maintenance. Such benefits promote adherence to long-term exercise programmes, which are a vital determinant of success with PD rehabilitation. Table tennis creates a social environment where participants can enjoy playing and aim for achievable progression within the sport, potentially leading to better engagement when compared with solitary exercises [16]. The satisfaction of skill progression and group-based play may encourage behavioural and neurological benefits.

Limitations With Existing Evidence

Despite the reassuring findings, the current literature available is restrained by methodological limitations. The pilot studies conducted by Olsson et al. [15] and Inoue et al. [18] were small-scale with limited generalisability. The control groups are inconsistently defined, and the small sample sizes, often less than 50 participants, do not prove generalisability to the broader population. The studies that look at table tennis as an intervention span up to six months in duration. For example, Dashtipour et al. [7] compared general exercise therapy with existing therapy in PD over a six-month period. Measured outcomes were considerably variable and often relied on subjective scales such as self-reported physical activity levels and diverse cognitive assessments. 

Additionally, the frequency, intensity, and format of table tennis sessions were not standardised in most studies. The variations in player experience, coaching, and physical adaptation to disease severity further complicate cross-study comparisons. There is potential bias introduced as there is a lack of randomised controlled trials and blinding of participants. Moreover, the absence of neuroimaging data limits the understanding of neural changes in response to interventions [30].

Another limitation is the insufficient quantity of studies that directly compare table tennis to already established interventions, for example, physiotherapy, Tai chi, dance, or boxing. Comparative data would enable the literature analysis to determine whether the advantages of table tennis therapy are unique due to its multimodal nature or if it mirrors the same effects of structured physical therapy.

Future Directions

Future research should prioritise large-scale, multicentre randomised controlled trials with standardised intervention protocols to strengthen the evidence base. Studies should consider including objective outcome measures, for example, gait analysis, neuroimaging, and posturography. Moreover, longitudinal studies assessing neuroprotective effects and long-term adherence can determine whether table tennis displays sustained benefits or slows disease progression.

The concept of the use of virtual reality technologies in exercise therapy presents another topic for further research. The 2025 Cochrane-registered multicentre randomised controlled study comparing virtual reality table tennis with conventional physical exercise in PD presents a scalable and accessible solution to improve adherence [31]. This approach may complement existing rehabilitation techniques by combining the elements of game playing with evidence-based motor and cognitive therapy. 

Finally, qualitative research can explore the patient experience, motivation, and barriers to participation [32]. This could aid the understanding of the psychosocial elements of adherence and well-being. Tailored programmes with adjusted intensities based on disease severity could enhance inclusivity.

Outcome

Current evidence collectively suggests that table tennis is a safe, feasible, and multimodal exercise for individuals with PD. The sport provides therapeutic benefits in motor, cognitive, and psychosocial domains. This may be mediated by mechanisms of neuroplasticity and cortical reorganisation. Preliminary findings are positive; however, the current literature remains limited by methodological variability and small sample sizes. Robust, standardised trials are required in order to establish the efficacy, training parameters, and long-term sustainability of table tennis as an intervention for PD. Given its accessibility, enjoyability, and holistic engagement of the sport, table tennis warrants integration into the Parkinson’s rehabilitation approach.

## Conclusions

Table tennis has emerged as a promising and feasible rehabilitation strategy for individuals with PD. Current evidence supports the benefits of motor, cognitive, and psychosocial outcomes through the therapeutic mechanism of neuroplasticity, cortical reorganisation, and sensorimotor integration. Table tennis creates an engaging, accessible, and social environment unlike traditional physiotherapy rehabilitation. This may enhance long-term adherence, which is a key factor for rehabilitation success. Although findings demonstrate positive outcomes, existing literature is limited by small sample sizes, methodological variability, and short intervention durations. Generalisability is further restricted due to the absence of standardised protocols, objective outcome measures, and comparative data with existing established exercise therapies. Future research is required with large-scale multicentre randomised controlled trials including a longitudinal follow-up and objective outcome measures. Virtual reality-based table tennis programmes may enhance accessibility and improve scalability.

To summarise, table tennis rehabilitation represents a holistic, enjoyable, and feasible strategy for PD therapy. The sport offers a combination of physical, cognitive, and social interaction, which is a useful addition to conventional exercise therapy when considering a multidisciplinary Parkinson’s model of care.
